# The effects of cGAS-STING inhibition in liver disease, kidney disease, and cellular senescence

**DOI:** 10.3389/fimmu.2024.1346446

**Published:** 2024-07-24

**Authors:** Ling Wang, Zhengwei Zhang, Haichao Zhang, Minmin Zhou, Cheng Huang, Wenjiang Xia, Jun Li, Hongmei You

**Affiliations:** ^1^ Department of Pharmacy, Shangyu People’s Hospital of Shaoxing, Shaoxing, China; ^2^ Inflammation and Immune Mediated Diseases Laboratory of Anhui Province, Anhui Institute of Innovative Drugs, School of Pharmacy, Anhui Medical University, Hefei, China; ^3^ Department of Pharmacy, Hangzhou Women’s Hospital, Hangzhou, China

**Keywords:** cGAS-STING, sterile inflammation, liver diseases, kidney diseases, cellular senescence

## Abstract

The cyclic GMP-AMP synthase (cGAS)-stimulator of interferon genes (STING) signaling pathway is one of the fundamental mechanisms of the body’s defense, which responds to the abnormal presence of double-stranded DNA in the cytoplasm to establish an effective natural immune response. In addition to detecting microbial infections, the cGAS pathway may be triggered by any cytoplasmic DNA, which is absent from the normal cytoplasm, and only conditions such as senescence and mitochondrial stress can lead to its leakage and cause sterile inflammation. A growing body of research has shown that the cGAS-STING pathway is strongly associated with sterile inflammation. In this study, we reviewed the regulatory mechanisms and biological functions of the cGAS-STING pathway through its involvement in aseptic inflammation in liver disease, kidney disease, and cellular senescence.

## 1 Introduction

A thorough summary of the many functions of the cyclic GMP-AMP synthase (cGAS)-stimulator of interferon genes (STING) pathway in malignancies and autoimmune disorders has recently been published. Nonetheless, a wealth of data suggests that the cGAS-STING pathway is strongly expressed in several non-immune-related tissues, including the liver (1), kidney (2), and lungs (3), in addition to most immune-related tissues. Endogenous chemicals activate cGAS-STING, which causes inflammation in various disorders. Oxidative stress and apoptosis cause mitochondrial DNA leaking in nonalcoholic fatty liver disease (NAFLD) (4), nonalcoholic steatohepatitis (NASH) (4), alcoholic liver disease (ALD) (5), and renal diseases (6); this “foreign” DNA is detected by cGAS-STING signaling and subsequently triggers the immune system. Both acute kidney injury and chronic kidney disease may be influenced by incomplete and functional mitochondria as well as mitochondrial DNA (mtDNA) copy number abnormalities (mtDNA-cn) (7). In response to cellular stress, mtDNA is released into the cytoplasm and detected by various DNA-sensing pathways, including cGAS-STING signaling (8). Compensatory increases in hepatic mitochondrial respiration, mitochondrial leakage, and hepatic STING protein levels have been observed in patients with NAFLD (9). Patients with alcohol use disorders and persistently alcohol-fed animals with an activated cGAS-STING pathway had considerably higher serum levels of mtDNA-enriched MPs (9, 10).

Cellular senescence, a permanent state of cell cycle termination, is one of the main causes of senescence-associated sterile inflammation and disease. It is primarily characterized by morphometabolic changes, permanent withdrawal from the cell cycle, resistance to cell death, and emergence of inflammatory senescence-associated secretory phenotypes (SASP) (11). The cGAS-STING pathway has been shown to be a significant signaling mechanism that stimulates SASP production in cells, and there is a substantial correlation between it and cellular senescence (12, 13). Because senescent cells express the nuclear marker lamin B1 (14), which is broken down by autophagy, the cytoplasmic DNA of these cells is derived from cytoplasmic chromatin CCFS. When lamin B1 autophagy brings DNA into the cytoplasm, cGAS recognizes the aberrant DNA and the cGAS-STING pathway is initiated, allowing cells to secrete SASP (15). Furthermore, β-amyloid buildup in the brains of patients with Alzheimer’s disease has been linked to double-stranded DNA (dsDNA) breaks and oxidative damage to mtDNA (16, 17). These events facilitate the release of mtDNA into the cytoplasm and activate the cGAS-STING pathway.

In this review, we emphasize the reaction of the cGAS-STING signaling mechanism to self-abnormal DNA and describe the current developments in kidney disease, liver disease, and cellular senescence.

## 2 cGAS-STING signaling molecular events occurring during immune responses

**Figure 1:**
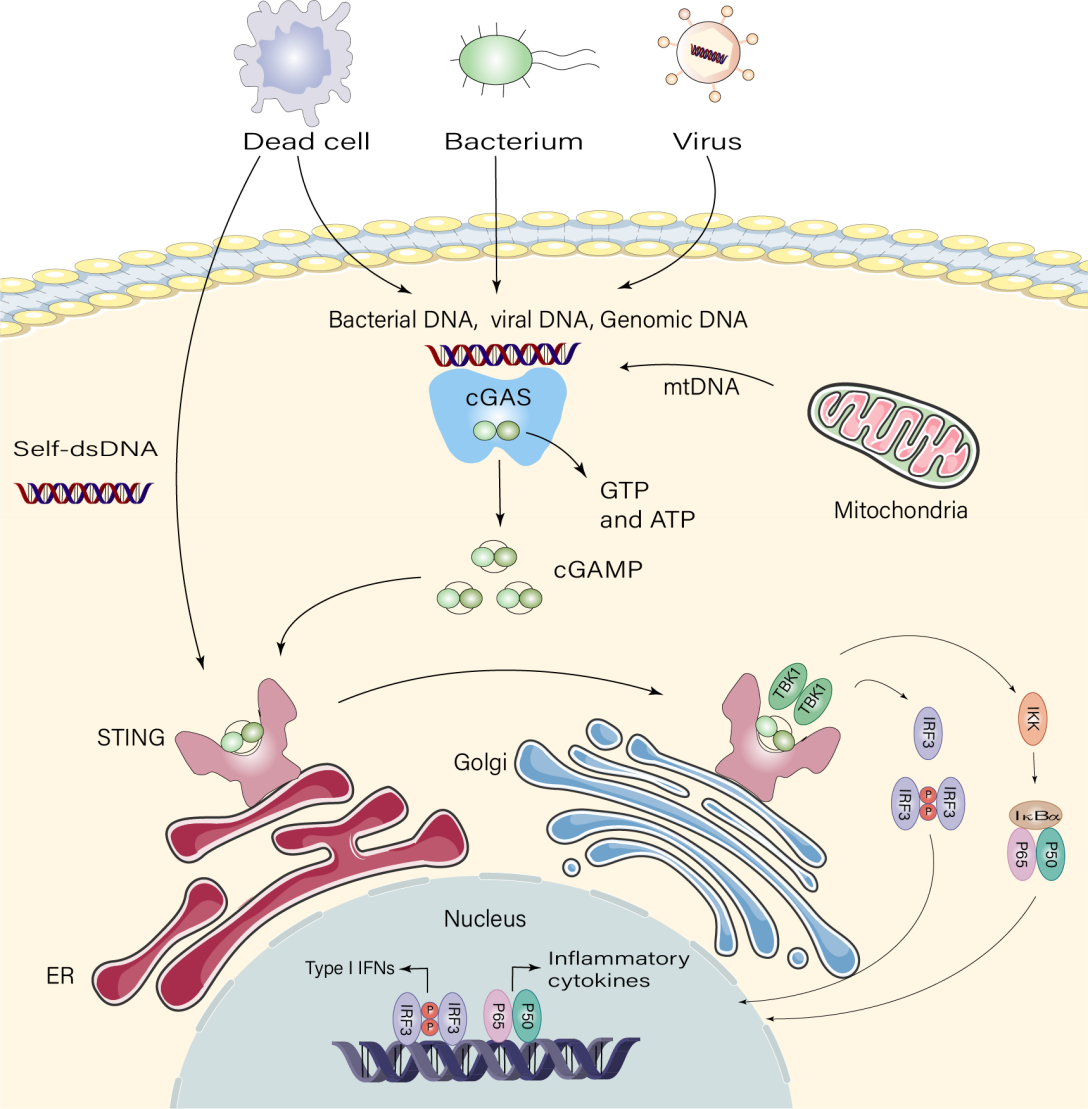
Signaling pathway of cGAS-STING. Dead cells, viruses, and damaged mitochondria release DNA that binds and activates the cyclic GMP-AMP synthase(cGAS), which catalyzes the synthesis of 2’3′-cGAMP from ATP and GTP. 2′3′-cGAMP binds to the endoplasmic reticulum adaptor STING, and cGAMP binds to the interferon gene-stimulating factor (STING) dimer on the endoplasmic reticulum (ER) membrane. STING then activates inhibitor of kappa B kinase (IKK) and TANK-binding kinase 1 (TBK1). TBK1 phosphorylates STING, which recruits TBK1, promoting autophosphorylation of TBK1, phosphorylation of STING, and recruitment of interferon regulatory factor 3 (IRF3). Phosphorylation of IRF3 by TBK1 dimerizes and translocates IRF3 to the nucleus, thereby inducing type I interferon, and gene expression of several other inflammatory mediators, pro-apoptotic genes and chemokines.

Recognition of foreign DNA is a crucial component of immunity in many species. The cGAS-STING pathway, which has emerged as a critical mechanism for connected DNA sensing and triggers innate immune defense programs, plays a major role in this process in mammalian cells (18). cGAS contains two primary DNA-binding structural domains and a nucleotidyl transferase structural domain. cGAS is self-repressed in the absence of DNA 2’ 3’ cyclic GMP-AMP (cGAMP) is produced when dsDNA binds to conjugated cGAS, activating its enzymatic activity (19). This isomer of cGAMP is known as “2’ 3’ ′-cGAMP” and functions as a second messenger by binding to the endoplasmic reticulum (ER) membrane adaptor STING (20, 21). STING proteins are activated by cGAMP synthase and move from the ER to the Golgi apparatus, where they undergo higher order zwitterionization to form tetramers (22). STING is directly activated by bacterial CDNs (23, 24). Interferon regulatory factor 3 (IRF3) and TANK-binding kinase 1 (TBK1) are believed to be recruited by palmitoylation of Golgi STING (25). STING tetramerization causes the TBK1 dimer to be recruited and activated, and TBK1 transphosphorylates STING’s c-terminal structural domain, activating IRF3 (26). Following its translocation to the nucleus, IRF3 stimulates the production of type I interferon (IFN-I) and immune-stimulated genes (ISG) (24). Additionally, STING may trigger inflammatory cytokines, including interleukin (IL)-6 and tumor necrosis factor (TNF), and activate nuclear factor kappa B (NF-κB) signaling (9) (**Figure 1**).

## 3 The role of cGAS–STING in liver disease

### 3.1 NAFLD

The spectrum of NAFLD includes a range of conditions, from simple accumulation of fat in the liver to inflammation, fibrosis, cirrhosis, and hepatocellular carcinoma (HCC) (27). NAFLD is divided into two subtypes: non-progressive non-alcoholic fatty liver (NAFL) and progressive non-alcoholic steatohepatitis (NASH) (28). These data suggest that NAFLD and NASH are promoted by the innate immune response. Therefore, it is unclear how the innate immune response contributes to NALFD at a fundamental level (29, 30). Obesity and NAFLD are strongly linked, and metabolic disorders are mostly influenced by persistent sterile inflammation of the adipose tissue (31). Chronic inflammation in adipose tissue is the primary cause of insulin resistance caused by obesity. Mouse adipose tissues showed significantly higher expression levels of cGAS and STING when the mouse were obese (31). In the mitochondrial matrix, the oxidoreductase-like protein (DsbA-L) functions as a chaperone-like protein. The targeted knockdown (KD) of this protein may compromise mitochondrial function and promote mtDNA release (32). A persistent sterile inflammatory response is triggered by the activation of the cGAS-STING signaling pathway by mtDNA escape generated by a high-fat diet (HFD). DsbA-L overexpression specific to the adipose tissue or STING KD shielded animals from obesity induced by a HFD. This suggests that by blocking the activation of the cGAS-cGAMP-STING pathway caused by mtDNA release, DsbA-L, a crucial regulator of mitochondrial integrity, shielded mice from inflammation and metabolic failure caused by obesity. This further implies that obesity-induced chronic inflammation may be ameliorated by focusing on the cGAS-cGAMP-STING axis in adipose tissue (32). The level of STING protein in the liver tissue of NAFLD patients also shows an increasing trend. (29). During the development of NAFLD/NASH, there is a notable increase in mtDNA levels in the blood and hepatocytes of humans and mice (33). One of the endogenous damage-associated molecular patterns, mtDNA, activates cGAS-STING signaling, which results in hepatocyte abnormalities (34). The STING-irf3 pathway connects the activated STING protein with Bcl-2-associated X protein (Bax) in the mitochondria, initiating the mitochondrial apoptotic pathway and resulting in apoptosis in an IFN-independent manner (35). IRF3 phosphorylation is inhibited by STING deficiency, and IRF3 phosphorylation is further inhibited by the absence of Bax interaction and activation of caspases 3 and PARP, which reduces hepatocyte death (36). In addition to apoptosis, inflammation is essential in the conversion of NAFLD to NASH, inflammation as well as apoptosis are essential (37). Activation of the hepatic cGAS-STING-TBK1 pathway may cause inflammation and interfere with cellular metabolism. These effects may include increased glycolysis and systemic lipid metabolism (38). Lipotoxic activation of TBK1 and consequent phosphorylation of p62 in hepatocytes are mediated by cGAS and STING. Accumulation of protein inclusions in hepatocytes is one of the main pathophysiological indicators of NASH. Large protein inclusions in hepatocytes and lipotoxicity-induced ubiquitinated protein aggregation depend on TBK1-mediated p62 phosphorylation (39, 40). Notably, Luo et al. (29) confirmed that plasma ALT and hepatic triglyceride levels were significantly lower in HFD wild-type (WT) mice than in HFD-fed STINGgt mice. In addition, hepatic inflammation, collagen deposition, and the expression levels of α-SMA and Col1α1 were significantly lower after inhibition of cGAS-STING compared to HFD-WT mice. The findings of Iracheta-Vellve et al (40). were also consistent. According to this research, a novel therapeutic target for stopping the onset and progression of NAFLD may be the cGAS-STING signaling pathway.

### 3.2 ALD and liver fibrosis

Hepatic inflammation, intestinal leakiness, and aberrant oxidative stress are associated with ALD, a global health concern (41). Numerous studies have indicated that sterile inflammation leads to alcoholic liver injury, and that lipogenesis and inflammation are both triggered by the cGAS-STING signaling pathway (42, 43). Hepatic RNA-seq analysis of patients showed a positive correlation between cGAS-IRF3 pathway expression and ALD severity (9). Connexin 32 (Cx32) plays a crucial role in ALD as a regulator of the 2’ 3’ cGAMP intercellular transfer produced by cGAS. Inhibition of Cx32 expression prevents the cytoplasmic sensor cGAS from inducing IRF3 activation in alcohol-damaged hepatocytes and the surrounding parenchyma via the gap junction intercellular communication pathway (44, 45). IRF3 has also been implicated in ALD. Petrasek et al. (46) reported that phosphorylated IRF3 and STING mediate ethanol-induced ER stress in hepatocytes during the early phases of ALD. Activation of IRF3, which is associated with the pro-apoptotic molecule Bax, results in hepatocyte death. Their results indicated that the first phases of ALD are driven by hepatocyte-specific IRF3 and not by macrophage-dependent inflammation, even though macrophages are required for the development of alcoholic steatohepatitis. IRF3 is independent of IFN-I and inflammatory cytokines, and it plays a unique role as a signaling molecule in the crosstalk between ER stress and hepatocyte death in ALD. To further validate the role of cGAS-STING in ALD, researchers conducted experimental observations. They found that mice with a STING gene knockout (KO) did not show obvious hepatocyte apoptosis after alcohol consumption compared to WT mice. In addition, these KO mice exhibited a reduced degree of fat degeneration and did not exhibit IRF3 upregulation. These results suggest that inhibition of the GAS-STING pathway has a significant protective effect against alcoholic liver injury and is of great significance for a deeper understanding of the mechanism underlying the development of this disease (46). Excessive build-up of the extracellular matrix (ECM) caused by long-term damage is known as liver fibrosis. Oxidative stress and inflammation accelerate liver damage (47). Without adequate therapy, liver fibrosis progresses to cirrhosis. Several causes of liver fibrosis have been documented for decades and several possible treatments have been suggested. However, there is currently no effective therapeutic strategy (48). TGF-β causes healthy HSCs to release mtDNA from voltage-dependent anion channels (VDACS) (49) and form an mtDNA cap on the outer membrane of the mitochondria, which activates the cGAS-STING-IRF3 pathway (50). Parkin is an essential regulator of mitochondria. Patients with liver fibrosis have elevated levels of circulating mtDNA, hepatic parkin, and VDAC1 (51). Parkin KD worsened hepatic mtDNA release and STING signaling activation and reduced autophagy and apoptosis in mice with liver fibrosis. Furthermore, mtDNA release into the cytoplasm was inhibited and VDAC1 oligomerization was stopped by parkin site-specific ubiquitination of VDAC1 at lysine 53. Liver fibrosis was inhibited by parkin-mediated ubiquitination of VDAC1 at certain locations, which stopped VDAC1 oligomerization and mtDNA release (52). Hepatocytes that accumulate microbial DNA also show high levels of phosphorylated STING and cGAS. Activation of the cGAS-STING signaling pathway leads to the overexpression of NLRP3 and enhances hepatic cellular pyroptosis, which is involved in liver fibrosis. In mouse models, acute and chronic fibrosis induced by carbon tetrachloride can be alleviated by the lack of STING, which reduces liver cell death, inflammation, and fibrosis (18). Furthermore, the effect of microbial DNA on the inflammatory response of insulin-sensitized cells was mitigated by cGAS KD (53). These results highlight the significance of Cgas-STING signaling in hepatocyte responses caused by microbial DNA. However whether the cGAS-STING pathway contributes to hepatic fibrosis by inducing cellular pyroptosis, reactive oxygen species (ROS) accumulation, or an inflammatory response needs to be further explored.

## 4 Link between cGAS–STING and kidney diseases

### 4.1 Acute kidney injury

High morbidity and mortality rates are associated with acute kidney injury (AKI). Elevations in serum creatinine levels and urine output were used to classify the severity of AKI (54). Acute kidney injury is a complex clinical condition that changes depending on the type of damage. However, the disruption of energy metabolism is shared by several acute renal injury disorders (54). Given that the energy metabolism pathways are important causes of AKI, they provide novel therapeutic options.

Similar to the elevated levels of STING observed in the kidneys of patients with AKI, the nephrotoxic drug cisplatin enhanced the production of cGAS and STING in injured tubules of mice. According to these results, the BCL-2-like protein 4 (BAX) hole in the outer mitochondrial membrane is the mechanism through which cisplatin promotes mtDNA leakage into the cell membrane (35). In a cisplatin-induced mouse model, tubular inflammation in AKI was activated via the cGAS-STING pathway, and in AKI mice, tubular inflammation was reduced by STING inhibition. By preventing mtDNA replication, the expression of the cGAS-STING pathway and cisplatin-induced inflammation were significantly reduced. According to previous studies, cisplatin exposure causes mitochondrial malfunction in tubular cells, which in turn leads to inflammatory reactions via cGAS-STING signaling induced by cytoplasmic mtDNA (6). The biological role of Myr, a flavonoid abundantly distributed in a variety of plants, was identified by Qi et al. (55). It was discovered that the HS15-Myr nanomicelles reduced cisplatin-induced AKI by blocking the cGAS-STING signaling pathway. By controlling mitochondrial processes, such as mitophagy and mitochondrial fission, PGAM5 plays an important role in cellular activity (56, 57). In the kidneys of mice with AKI and human renal biopsy samples, PGAM5 levels were elevated. PGAM5 dephosphorylates Bax, which causes mtDNA leakage, which is linked to renal injury and the inflammatory response in AKI. To reduce the inflammation in AKI, PGAM5 KD prevented the release of mtDNA and the interaction of the cellular DNA receptor cGAS with mtDNA in cultured renal tubular epithelial cells (2). cGAS-STING KO by small interfering RNA resulted in a cisplatin-induced decrease in the phosphorylation of the downstream target proteins TBK1 and P65 in HK-2 cells, which ameliorated renal tubular inflammation and the extent of neutrophil infiltration in the tubular interstitium in mice with AKI (6). This suggests that the activation of the cGAS-STING pathway plays an important role in acute kidney injury. However, in an AKI mouse model, STING KO did not completely eliminate damage or inflammatory responses, indicating that upstream mitochondrial dysfunction may lead to the activation of other downstream pathways that participate in cisplatin-induced AKI.

### 4.2 Chronic kidney disease

Chronic kidney disease (CKD) is caused by inflammation, cellular and ECM deposition, and nephron loss (58). However, persistent activation of this pathway worsens chronic kidney disease and may result in renal cancer (59).

A mouse model of CKD showed that the renal cortex and glomeruli activate the cGAS-STING signaling pathway. Mitrofanova et al. employed terminally differentiated murine and human podocytes as *in vitro* models to examine the presence and function of various elements involved in the cGAS-STING signaling pathway (60). For *in vivo* investigations, they employed db/db mice as a representative model of type 2 diabetes (T2D) accompanied by diabetic kidney disease (DKD), and Col4a3-/- mice as an experimental model for kidney disease linked to Alport syndrome (AS). The results indicated that the cGAS-STING signaling pathway was expressed in both human and murine podocytes. Proteinuria and podocyte loss were observed in WT mice following STING activation. In addition, activation of the cGAS-STING signaling pathway was observed in both the kidney cortices and glomeruli of db/db mice and mice with AS. Furthermore, this activation was correlated with the presence of albuminuria. Notably, genetic or pharmacological suppression of STING effectively improved glomerular injury and kidney failure induced by diabetes or streptozotocin as well as AS-associated conditions (60). Further research is necessary to determine the exact mechanisms underlying this phenomenon. Innate immunological protection against infection is facilitated by the cGAS-STING signaling pathway, and controlling the degree of STING suppression is crucial for the management of CKD.

DKD is a common complication that typically develops in patients several years after diabetes onset (60). DKD causes serious damage to the kidneys, including decreased glomerular filtration rate, proteinuria, and ultimately, kidney failure. Although there are currently some treatments for DKD, such as interventions to control blood pressure, blood sugar, and lipid levels, additional treatment targets and methods are needed to effectively manage this severe complication. In recent years, increasing evidence has shown that chronic low-grade systemic inflammation and oxidative stress play key roles in the development of DKD (61). Previous studies have shown that an increase in PCSK9 is associated with renal inflammation in mice fed a HFD, and with HGPA-induced inflammation in HK-2 cells. The cGAS/STING pathway is involved in the development of DKD. Further experiments showed that PCSK9 triggered mtDNA damage in DKD and activated the cGAS-STING pathway, leading to a series of adverse immune reactions and renal tissue damage. The use of the STING inhibitor C-176 in renal tubular epithelial cells HK-2 significantly reduced the expression levels of p-TBK1/NF-κB, thereby alleviating kidney inflammation caused by chronic kidney injury and immune-mediated kidney injury (62). STING KO mice have also been studied. WT and STING KO mice were used to establish a streptozotocin-induced diabetes model. Compared to the WT mice in the STZ group, the increase in the levels of urinary albumin and urinary Kim-1 was significantly reduced in the STING KO mice with neuropathic pain (63). These results suggest that the cGAS-STING/p-TBK1/NF-κB axis plays a key role in the development of diabetic nephropathy. In conclusion, these findings provide new insights for the treatment and prevention of immune-related chronic kidney damage, and provide important references for further exploration of the mechanism of action of the cGAS-STING/p-TBK1/NF-κB axis in other immune-related diseases.

Various types of kidney disorders are associated with tissue damage, which has been primarily attributed to the effect of IFN-I, a cytokine widely employed in the field of clinical oncology for many years, (64) on multiple renal cell types, thereby influencing their biology and function. Augmented IFN-I signaling is detected during viral infections and autoimmune conditions, all of which may contribute to kidney impairment and share histopathological and pathogenic characteristics (65). It is worth noting that STING pathway activation serves as a pivotal mechanism for sensing innate immunity, resulting in the production of IFN-I within the tumor microenvironment. As CD8+ T cells are associated with tumors exhibiting an IFN-I signature, the potential for utilizing intratumoral STING agonists as a viable cancer treatment shows promise (66). The host STING pathway is at the interface of cancer and immunity. Activation of the STING pathway in the tumor microenvironment by antigen-presenting cells results in the generation of IFN-I and facilitates the spontaneous development of CD8+ T-cell responses against tumors, which exhibited therapeutic effects in multiple murine cancer models (67, 68). Xing et al. (69) reported that TRIM29, a protein abundantly present in human AECs and induced in myeloid dendritic cells upon exposure to dsDNA, is utilized by DNA viruses such as the Epstein-Barr virus, which inhibits the innate immune response of the host (70). Previous studies have indicated that TRIM29 acts as a crucial suppressor of the innate immune response to DNA viruses by specifically interacting with STING, thereby facilitating the prolonged presence of DNA viruses (70). Similarly, Fang et al. reported that the suppression or removal of TRIM18 in macrophages from humans or mice resulted in an increased production of IFN-I when exposed to dsRNA and dsDNA, as well as during infection with RNA and DNA viruses. From a mechanistic standpoint, this study revealed that TRIM18 plays a role in recruiting PPM1A to dephosphorylate TBK1. Deactivation of TBK1 prevents its interaction with upstream adaptors of mitochondrial antiviral signaling and STING, ultimately leading to the attenuation of antiviral signaling during viral infections (71). Interestingly, a study conducted in animals has provided evidence suggesting the involvement of TRIM18 in kidney damage and fibrosis in mice with diabetes (72). Inhibition of TRIM18 has been shown to effectively prevents inflammation, epithelial-mesenchymal transition, and fibrosis in HK-2 cells induced by high glucose levels (72). Previous research has indicated that TRIM29 facilitates podocyte pyroptosis via the NF‐kB/NLRP3 inflammasome signaling pathway and aggravates diabetic nephropathy (70). However, increasing evidence has revealed that pyroptosis is a double-edged sword in human cancers. Cancer cell pyroptosis contributes to the suppression of tumor progression (73), whereas pyroptosis in normal tissues contributes to the side effects of chemotherapy. Thus, further validation of the upstream and downstream relationship between TRIM29/TRIM18 and STING in various renal diseases is necessary to determine whether TRIM29/TRIM18 can effectively regulate STING, thereby controlling acute and chronic kidney injury, as well as renal cancer. However, it is imperative to further investigate the extent and proportion of pyroptotic cell death mediated by TRIM29 in both normal kidney and renal cancer cells. Undoubtedly, this remains a substantial undertaking requiring gradual and comprehensive exploration.

## 5 Signaling and regulation of cGAS-STING in cellular senescence

### 5.1 cGAS-STING in age-related diseases

Irreversible cell-cycle arrest, known as cellular senescence, restricts the ability of injured cells to proliferate (74). Genetic instability is a defining feature of the aging process (75). The SASP, which is characterized by the secretion of pro-inflammatory cytokines, chemokines, the ECM, and growth regulators by senescent cells, has recently been demonstrated to be induced by DNA damage (76). cGAS-STING promotes inflammatory senescence by increasing SASP secretion (12). This indicates a surprising relationship between the two seemingly unconnected channels.

Senescent cells are subtly influenced by the SASP to undergo self-destruction and cause neighboring cells to undergo senescence. As a result, age-related illnesses such as Alzheimer’s disease (AD) can be affected by cellular senescence caused by the SASP (77). AD is a neurodegenerative disorder. The condition is defined by the buildup of amyloid β peptide (Aβ) plaques in the brain, the tangle of phosphorylated Tau proteins and neurogenic fibers, and gradual cognitive deterioration in patients (78). Damage to mtDNA, the only organelle with its own genome, is linked to several neurodegenerative illnesses. Unlike genomic DNA, the mitochondrial genome is far more vulnerable to oxidative damage and lacks a strong defense mechanism against damage (12). Consequently, clearance of damaged mitochondria by autophagy is crucial (79). However, in the brains of AD model mice, the interaction between cGAS and dsDNA increased more than 4-fold compared to WT animals because of defective mitochondrial autophagy in senescent cells (80). Furthermore, the brains of patients with AD and senescent animals exhibit markedly increased levels of STING, TBK1, and IRF3 phosphorylation, suggesting that the cGAS-STING pathway is activated (77). In the brain, microglia and astrocytes operate as glial cells, promoting the survival and functionality of neuronal cells, exhibiting neurotrophic effects, and upholding the overall homeostasis of the brain (81). However, in illnesses, the inherent homeostatic qualities of glial cells are jeopardized, releasing inflammatory mediators that create a harmful inflammatory brain environment (21). Microglia, astrocytes, and cortical neurons all exhibit activation of the cGAS-STING pathway; however, only microglia show signs of inflammation. Activated microglia in AD model mice generated IL-1β and TNF-α, which changed astrocytes into neurotoxic A1-type astrocytes (81). ROS, reactive nitrogen species (RNS), and pro-inflammatory cytokines are produced in concert by activated astrocytes and microglia. Inducible nitric oxide synthase (iNOS) causes RNS. Nitrosative stress and the activation of additional neurodegenerative pathways are caused by elevated levels of nitric oxide and its highly reactive derivative peroxynitrite (82). cGAS KO in the brains of AD mice with reduced Aβ levels and a better proportion of microglia near Aβ plaques suggests that deletion of cGAS promotes Aβ clearance by microglia and ameliorates AD-associated cognitive decline. Deficiency of cGAS in Aβ42 peptide-treated primary microglia leads to the inhibition of the cGAS-STING pathway, decreased phosphorylation levels of TBK1 protein, and reduced expression of IFNβ, CXCL10, and CCL5. This also results in the inhibition of neurotoxic A1-type astrocytes, thus alleviating the brain damage caused by Aβ. In addition, *in vitro* experiments have shown that cGAS inhibitor RU.521 and STING inhibitor H-151 can protect neurons from Aβ damage; *in vivo* experiments have demonstrated that they significantly reduce the levels of Aβ in the brain and decrease the number of activated microglia and astrocytes, thereby improving cognitive decline associated with AD. These results provide important clues for finding new approaches to treat AD, and offer valuable information to gain a deeper understanding of the pathogenesis of AD. Hopefully, through further research and exploration, more effective and safer treatment methods can be developed to address this common but incurable neurodegenerative disease.

**Figure 2:**
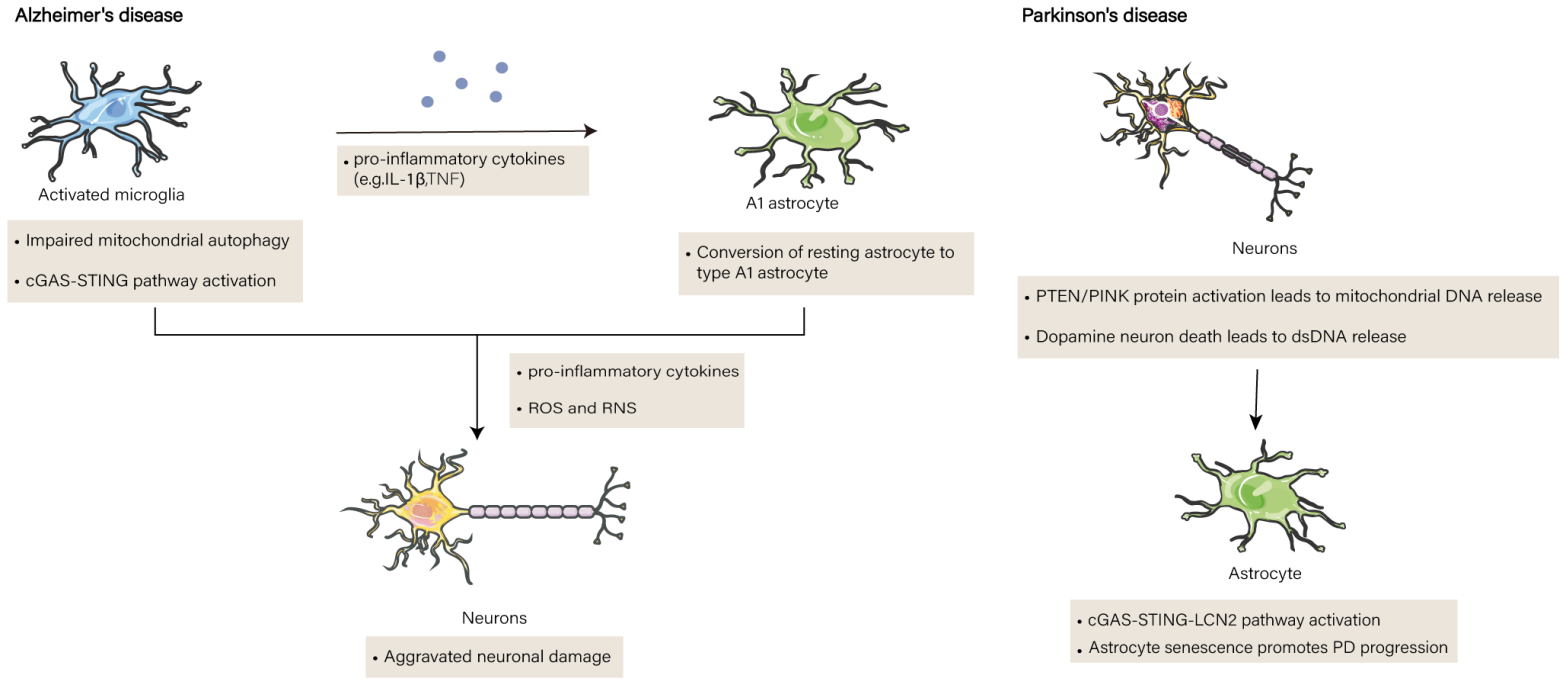
The function of cGAS-STING in brain diseases. Impaired mitochondrial autophagy in senescent cells in the brains of Alzheimer’s patients results in the release of mitochondrial DNA (mtDNA) into microglia, where the cGAS-STING pathway on microglia is activated and leads to the release of pro-inflammatory factors. Pro-inflammatory factors induce the transformation of astrocytes into type A1 astrocytes. The two cells synergize to produce pro-inflammatory cytokines, reactive oxygen species (ROS) and reactive nitrogen species(RNS), ultimately leading to neurodegeneration. In addition to the release of double-stranded DNA (dsDNA) from dead dopamine neurons in Parkinson’s disease mice, an increase in phosphatase and tensin homolog/PTEN induced putative kinase 1 (PTEN/PINK1) proteins induces the release of mtDNA from the voltage-dependent anion channel (VDAC) and bcl2-associated X protein/bcl2 antagonist/killer (BAX/BAK) channels, both of which activate the cGAS-STING signaling pathway, and the STING proteins increase the transcription oflipocalin 2 (LCN2), thereby secretion of senescence-associated secretory phenotype(SASP), and contribute to the aggravation of senescence in astrocytes. and promote astrocyte senescence and aggravate the disease process.

In addition to AD, Parkinson’s disease (PD) is significantly influenced by cGAS-STING in glial cells (83). Damaged dsDNA produced by dying dopaminergic neurons stimulates cGAS/STING (84). PD is characterized by an inflammatory response resulting in dopaminergic neuronal damage and death. et al. Barazzuol et al. (85). discovered that a mutation in the protein parkin, phosphatase, and tensin homolog (PTEN)-induced kinase (PINK) catalyzes a burst of Mfn2 phosphorylation. This in turn causes the Mfn2 complex to break down from the outer mitochondrial membrane in a p97-dependent manner, separating the mitochondria from the ER and promoting mitochondrial autophagy (86). The VDAC pore of the mitochondrial outer membrane allows the release of mtDNA and mediates activation of the cGAS-STING pathway. Alternatively, mtDNA can protrude through the inner membrane of the mitochondria and be released by the BAX/BAK complex (87). When cGAS-STING is activated in astrocytes, p16INK4a and p-p65 expression are upregulated, and STING proteins directly bind to YY1, facilitating YY1 nuclear translocation and boosting LCN2 transcription (88). Increased SASP secretion accelerates neurodegenerative lesions, and cGAS-STING-LCN2 stimulates senescence-associated β-galactosidase activity and astrocyte senescence, making them larger and flatter (89) (**Figure 2**). The loss of cGAS in PD mice leads to the blockade of LCN, and a significant downregulation of P161NK4a, p-STING, and p-p65 expression, while significantly improving the behavior of MPTP-treated mice and reversing dopamine (DA) levels in PD model mice. These findings indicate that cGAS plays an important role in the development of PD.

### 5.2 cGAS-STING in atherosclerosis

Atherosclerosis (AS) is characterized by accumulation of vascular smooth muscle cells (VSMCs), vascular plaque formation, and cholesterol overload (macrophage accumulation). Additionally, senescence of VSMCs is linked to the formation of micronuclei, activation of cytoplasmic sensing by cGAS-STING, and induction of multiple pro-inflammatory cytokines (90). During AS, VSMCs exhibit signs of senescence and DNA damage. IFN-I is induced by IRF3, nuclear DNA in the cytoplasm triggers the cGAS-STING cytoplasmic DNA sensing pathway, and NF-κB triggers a pro-inflammatory response (91). The activation of the TBK1 and cGAS-STING pathways is linked to this pro-inflammatory phenotype, as cGAS deletion eliminates the induction of IL-1α and IL-8 and raises NF-κB phosphorylation. Furthermore, IQ motif-containing GTPase-activating protein 1 (IQGAP1) functions as a scaffolding protein that controls mitochondrial activity and encourages the generation of ROS, which results in oxidative stress and mitochondrial malfunction, and is linked to the development of atherogenesis (92). Induction of mtDNA release into the cytoplasm and activation of the DNA sensor cGAS-STING to trigger endothelial cell pyroptosis to generate AS are the results of ROS generation promoted by IQGAP1. Moreover, TDP43, a nuclear (DNA)/RNA-binding protein that has been demonstrated to be localized to the mitochondria, increases the production of SASP by releasing mtDNA and initiating the cGAS-STING signaling pathway (93). The use of the cGAS inhibitor RU.521 and STING inhibitor C-176 in human umbilical vein endothelial cells reversed IQGAP1-induced elevated levels of NLRP3, caspase-1, and inflammatory cytokines (92). Moreover, studies have found that a HFD leads to increased expression of cGAMP and STING in ApoE-/- mice. Treatment of mice with the specific STING antagonist C-167 reduced plaque damage and decreased lipid accumulation. Additionally, stimulation-induced downstream molecules IRF3 and various typical inflammatory markers such as IL-6, IL-1β, and TNF-α were found to be elevated in AS plaques (94). Genetic associations in humans suggest that cellular senescence plays a protective role against AS (95). This suggests that senescence may play a dual role: the growth of atheromatous plaques is limited by macrophage and monocyte proliferation blockade; however, the disease process can also be induced by cell-secreted SASP factors (96).

### 5.3 Functions and signaling pathways of cGAS-STING in aging

Cellular senescence is closely associated with aging and age-related diseases. While youthful stromal cells regenerate senescent stem cells and the senescent milieu “ages” young stem cells, the accumulation of senescent cells is the primary cause of senescence-associated tissue deterioration. Cells use a process called mechanical signal transduction to detect the structural, mechanical, and physical characteristics of their surroundings. Guarnieri et al. (97) discovered that by blocking the cGAS-STING signaling pathway, stromal cells with intact YAP/TAZ mechanotransduction may postpone senescence. SASP and IFN-I-stimulated genes were secreted in greater amounts by WI-38 fibroblasts when YAP/TAZ was knocked down; however, the levels of these genes were reversed upon cGAS/STING KD. Both young and old YAP/TAZ-KO animals had noticeable and severely malformed nuclei, and the absence of YAP/TAZ caused an increase in cGAS at cell nuclear membrane discontinuities. YAP/TAZ mediates suppression of SASP gene expression and cGAS-STING activation by shielding the shell membrane via ACTR2 and lamin B1. Furthermore, the serine/threonine kinase,PINK1, regulates mitochondrial dysfunction (98). Renal tubular injury and an increase in senescence and SASP are the results of PINK1 deficiency (99). In the renal tubular epithelial cells of 24-month-old Pink1^-/-^ mice, these abnormalities were particularly noticeable. According to a gene expression study by RNA sequencing, PINK1 deficiency was linked to an elevated inflammatory response, and the activation of cGAS-STING was noteworthy in 24-month-old Pink1^-/-^ mice. Pink1 deficiency has been linked to mitochondrial malfunction, which can activate the cGAS-STING pathway and induce aging-related inflammatory responses. Pink1 is also associated with renal aging (86).

**Figure 3:**
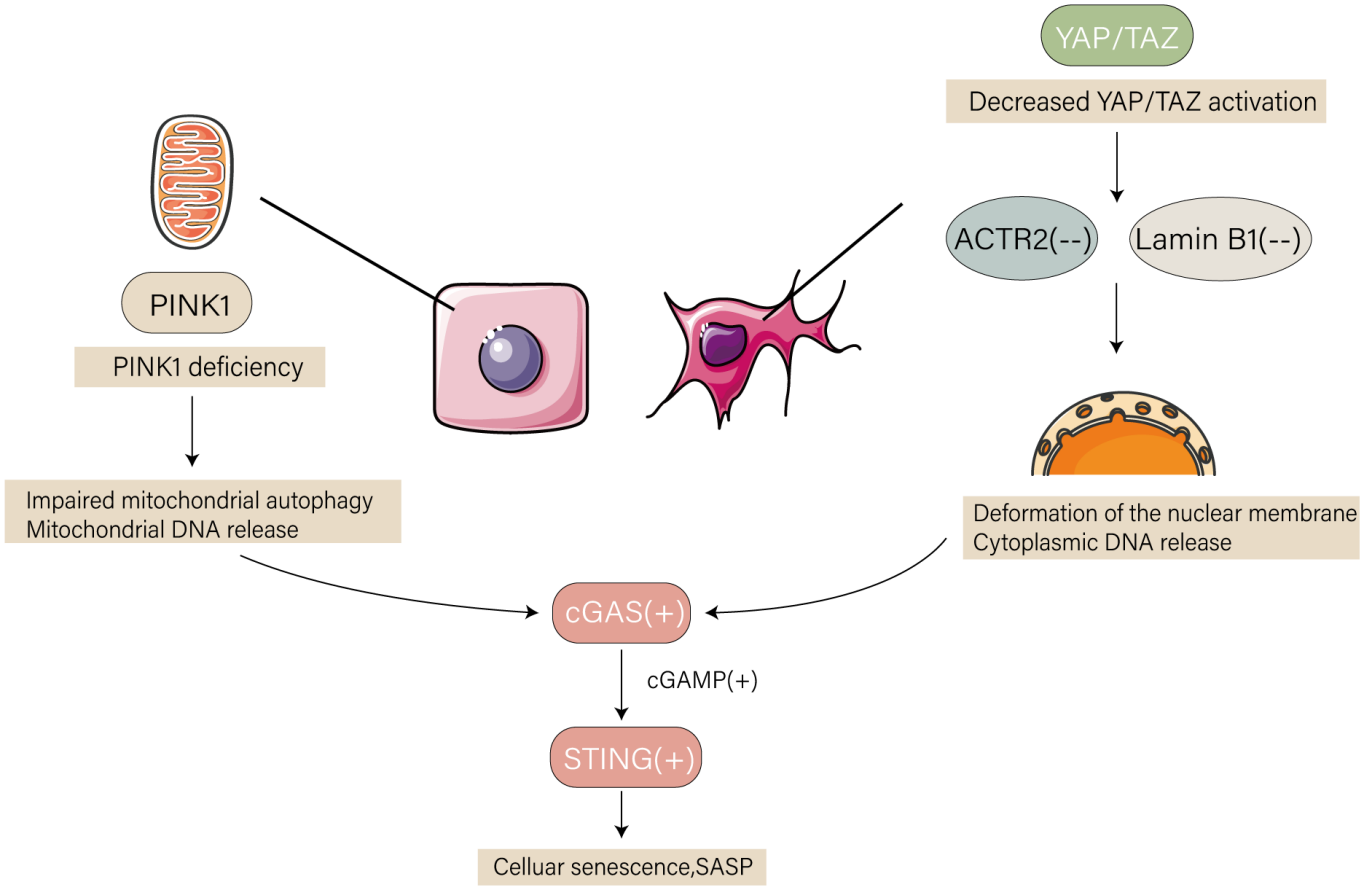
Signaling pathways upstream of cGAS-STING in aging. Deletion of yes-associated protein/transcriptional coactivator with pdz-binding motif (YAP/TAZ) protein in the nucleus of mouse dermal fibroblasts inhibits laminB1 and actin related protein 2 (ACTR2) proteins at the nuclear membrane, leading to nuclear deformation and leakage of nuclear DNA; deletion of PINK protein in the mitochondria of senescent mouse renal tubular cells leads to mitochondrial autophagy damage and leakage of mitochondrial DNA. DNA binds to cGAS to produce cGAMP, and STING binds to cGAMP, part of which interacts with protein kinase R-like endoplasmic reticulum kinase (PERK), leading to significant phosphorylation of el2α and inhibition of the cap-dependent mRNA translation process, which specifically promotes part of inflammation and cellular senescence. The other part is involved in cellular senescence by activating TBK1, which secretes SASP through the classical pathway.

The non-traditional cGAS-STING signaling pathway plays a crucial role in cellular senescence, in contrast to the classical cGAS-STING pathway. Significant phosphorylation of the elF2a S51 site and inhibition of cap-dependent mRNA translation processes can result from the activation of cGAS-STING signaling during cellular senescence. This function is independent of the classical STING complex, which consists of the key kinase TBK1 and signaling protein IRF3, in various species (100). Consequently, elF2α is phosphorylated by PERK in response to DNA sensing, which greatly reduces mRNA translation across the cell but specifically increases the inflammatory translational program. Parallel to but much earlier than the traditional STING-TBK1-IRF3 signaling in the Golgi apparatus, STING-PERK-elF2α signaling is immediately triggered in the ER. Taken together, these findings show that the cGAS-STING pathway plays a critical role in the regulation of cellular senescence, and that blocking this pathway might be beneficial for treating disorders associated with cellular senescence (**Figure 3**).

## 6 Conclusion and future directions

In this review, we provide an overview of the current developments in our understanding of the function of the cGAS-STING pathway in kidney, liver, and cell senescence disorders. In addition, the sterile inflammatory SASP, which is a characteristic of senescence and contributes to aging, is related to cGAS-STING. This contrasts with the inflammation that arises in response to pathogens such as bacteria and viruses. Finally, while pathway inhibitors of cGAS-STING are an effective way to treat chronic inflammation, the diversity of endogenous cytoplasmic DNA functions must be considered. For example, inhibition of CCF activation downstream of cGAS-STING signaling in chronic inflammation may be beneficial during aging, but it may also impair micronuclei-initiated cell-intrinsic immune detection and tumor suppressor signaling. Overall, these studies provided new ideas and targets for the treatment of aging and metabolic diseases. Therefore, the development of cGAS and STING proteins has received considerable attention in recent years. Several targeted drugs are currently undergoing clinical trials and are expected to be used for disease treatment in the future.

GTPase-activating protein SH3 domain-binding protein 1 (G3BP1) is a key protein in the sensing and activation of cGAS with DNA, and its deletion causes inefficient binding of cGAS to DNA and inhibits cGAS-dependent IFN production (101). Liu et al. (102) found that the natural small molecule epigallocatechin gallate (EGCG), identified in green tea polyphenols, could interfere with the binding of G3BP1 to cGAS, thus preventing cGAS from recognizing abnormal DNA and resulting in the inability of downstream pathways to be activated. The effectiveness of EGCG inhibitors has been demonstrated in animal models of autoimmune diseases and in patients with Aicardi-Goutières syndrome (AGS). The team also found that in the cells of patients with AGS as well as in mouse models, aspirin acetylated lysine residues (Lys384, Lys394, and Lys414) at positions 384, 394, and 414, respectively, of cGAS, which kept the cGAS protein inactive, prevented cGAS from activating downstream signaling pathways, and inhibited autoimmune responses (103). Recently, Chu et al. (104) reported that perilla aldehyde inhibits the auto-intrinsic immunity induced by cytoplasmic DNA by inhibiting cGAS activity. Mice treated with perilla aldehyde – a natural monoterpene extracted from Perilla frutescens – in the AGS disease model showed reduced expression levels of several ISG, such as Cxcl10, Isg15, Isg56, and Ifit3, and significantly attenuated autoimmune inflammation. Existing chemicals can serve as valuable bases for the development of clinical drug candidates. cGAS-STING-mediated sterile inflammation in liver and kidney diseases and aging warrants further exploration in clinical settings.

## Author contributions

JL: Conceptualization, Supervision, Writing – review & editing. LW: Writing – original draft. ZZ: Writing – review & editing. HZ: Supervision, Writing – review & editing. MZ: Supervision, Writing – review & editing. CH: Writing – review & editing. WX: Conceptualization, Supervision, Writing – review & editing. HY: Conceptualization, Supervision, Writing – review & editing.
